# Cancer Awareness and Screening Practices of Ghanaian Adults: A Cross‐Sectional Survey

**DOI:** 10.1002/cnr2.70144

**Published:** 2025-02-09

**Authors:** Patrick Kafui Akakpo, Martin Gameli Akakpo

**Affiliations:** ^1^ Department of Pathology ‐ School of Medical Sciences University of Cape Coast Cape Coast Ghana; ^2^ Department of Human Development and Psychology ‐ Faculty of Arts and Sciences Regent University College of Science and Technology Accra Ghana

**Keywords:** awareness, cancer screening, determinants, online survey, prevention

## Abstract

**Background:**

Cancer screening has been identified as an important contributor to cancer prevention and the control of both morbidity and mortality from cancer. Despite its importance, screening rates have remained low in Ghana. This study investigated some key predictors of screening habits and the rates of awareness for selected cancers that are amenable to screening and early detection. The health belief model provided theoretical support for the investigation.

**Methods:**

Data was collected from 503 adults in an online survey with a questionnaire, between June and August 2021. Univariate statistical analysis was used to determine the frequencies and percentages of variables. The multivariate analysis used a correlation and a logistic regression to measure association and test a model.

**Results:**

Participants were aged between 18 and 74 with a mean age of 32.74. Females made up 61.4% of the sample while males accounted for 38.6%. Only 37.6% of participants had previously screened for cancer while 62.4% had never screened. The study hypothesized that age, gender, and Cancer Awareness predict the Cancer Screening habits of respondents. The logistic regression showed that, Age (*B* = 0.10, SE = 0.01, *p* = 0.00) and Gender (*B* = −0.2.71, SE = 0.30, *p* = 0.00) predicted cancer screening habit.

**Conclusion:**

Age and gender can predict screening habits. Awareness did not predict screening in this study. The reason and meaning of the findings are discussed and suggestions for improvement of screening uptake and for future research are provided.

## 1 Introduction

Cancer screening is recommended by the WHO and major healthcare stakeholders to prevent and control rates of morbidity and mortality related to cancer. Screening habits in Ghana are generally below recommended levels and the burden of cancer care on the healthcare budget is increasing. More cancers are detected in developed countries than in developing countries like Ghana, but morbidity and mortality rates in Ghana are higher and usually underreported [1]. The leading causes of cancer mortality in Ghana include cervical cancer and breast cancer for women and, prostate and hepatocellular carcinoma for men [2]. The World Health Organization's Global Cancer Observatory data for Ghana [2], recorded 27, 385 new cases in 2022 with a worsening outlook reported by several studies such as Zoure et al. [3], Mensah and Mensah [4], and Spearman et al. [5].

Most patients are diagnosed late despite evidence that some of these cancers are preventable and early detection improves outcomes. Screening is key to reducing morbidity and mortality from cancer in Ghana, especially since there are only three centers across the entire country that can provide radiation and other advanced oncological services [6]. The identification of the importance of cancer screening in this regard and the knowledge of the burden of disease from some cancers such as cervical cancer informed the decision to include screening for cervical precancer in the National Health insurance scheme of Ghana.

This agrees with a wide body of research that has identified screening as important for cancer control [6, 7].

However, despite numerous campaigns focused on breast [8], cervical [9], and prostate [10] cancers, screening has remained generally low [6, 7].

Our study investigated the level of cancer awareness, screening rates, and predictors of the likelihood of cancer screening in adults with the aim of gaining insights into knowledge and behavior as it relates to cancer screening in general. This is based on our belief that individuals are more likely to undergo cancer screening when they understand and value its benefits.

Previous studies have predominantly focused on breast, cervical, and prostate cancer [1, 9]. This has generally left out knowledge about several other cancers that can be screened for and are of equal importance in Ghana [11]. This paper thus included colon, ENT (ear, nose and throat), gastric and liver cancers to provide insights into public awareness of these other cancers.

In Ghana, cancers are mostly diagnosed at an advanced stage with reduced chances of cure or prolonged survival [4, 12]. This also increases the burden on both patients and caregivers [13]. Screening is therefore a useful habit, which increases the probability of early detection and treatment aimed at achieving a cure [4].

The current high mortality rates and late presentation in patients has partly been attributed to low awareness amongst people in developing countries including Ghana [14]. O'Mahony et al. [15] noted that awareness constitutes an understanding of symptoms, demographic risks, environmental and genetic associations, and the benefits of mitigating behaviors. Demographic risks include age and gender which increase the probability of specific cancers. Some recent awareness campaigns in Ghana have primarily focused on gender and age‐related risk factors. Gender related campaigns have promoted breast self‐examination and PAP smear tests for females. Males have been advised to seek prostate cancer screening from the age of 40 in certain campaigns. In line with this there have been several cancer awareness campaigns that highlight the role of oncogenic viruses in the causation of cervical cancer, hepatocellular carcinoma, ENT cancers, and penile cancers. Most of these oncogenic viral infections are preventable through vaccination and long‐term screening. Cancer screening, aimed at detecting cancerous lesions or early localized cancer has been identified as an important contributor to cancer prevention, early detection, and lower morbidity and mortality. Follow‐up treatment can then be localized and effective, resulting in complete cure and ensuring long‐term survival. Screening is thus an important activity in any comprehensive cancer control program. According to Ampofo et al. [9], the rate of cervical cancer screening in Ghana is low, with factors such as low educational level, unmarried status, and unemployment predicting non‐screening among women. In a study, Ebu et al. [6] report that only 0.8% of patients in the cohort had ever screened for cervical cancer. The low level of awareness is a barrier to regular screening because of the inability of many individuals to perceive the risk associated with late‐stage presentation [16].

### 1.1 Rationale for the Study

The rationale for our study was to test the influence of cancer awareness, age, and gender on screening habits for a diverse pool of cancers. Previous studies have focused on the most well documented cancers, and this has created a gap for evidence to drive studies and programs about other cancers, specifically, colon, liver, ENT and, gastric cancers. Evidence from the study can improve cancer awareness and screening promotion campaigns by addressing a broad range of cancers, which are all a burden to the Ghanaian health system.

### 1.2 Objectives

Based on the previous findings [4, 6, 7, 15] about the behavior modifying roles of awareness, age and gender, the study tested the following hypotheses.

1. Gender will predict screening habits for Cancer.
2. Age will predict screening habits.
3. Awareness will predict screening habits

### 1.3 Theory

The Health Belief Model (HBM) is used as the theoretical framework for this study. The HBM, a social‐psychological theory from a social cognitive perspective, was developed in the United States in the 1950s to help predict preventive health behavior. The model was used by Hochbaum [17] in a 1956 study of X‐Ray screening for Tuberculosis. At the time of Hochbaum's study, there was evidence that some TB patients could be asymptomatic and thus not show any outward signs of infection. Screening was promoted as a safe way to detect infection rates early and reduce its spread amongst the population. In Hochbaum [17], perceived susceptibility of the patient to TB and the perception that screening is beneficial, predicted their participation in screening programs. Rosenstock [18] noted that several studies followed with the aim of using the HBM ideas to increase perceptions of susceptibility and improve awareness about the benefits of screening. In recent years, self‐efficacy has been included in the model to account for task specific self‐confidence [19].

Key components of the HBM include perceived susceptibility, perceived severity, health motivation, perceived benefits, and perceived barriers [20]. These perceptions and motivation are influenced by demographic and psychological variables [19]. The action taken by the individual is predicted in a manner reflecting their perceptions and motivation. Susceptibility and severity deal with perceptions of risk and the evaluation of seriousness associated with a consequence. Health motivation refers to the concern an individual demonstrates about healthcare topics. The perceived benefits in the HBM relate to the subjective idea that an action like cancer screening reduces severity or threats. The perceived barriers mentioned in the model focus on disadvantages of acting. The “desirable” behavior to be predicted by all these perceptions and motivation is termed “action” which is influenced by cognitive prompts or cues to act to increase the perceived benefits. These can range from regular screening to regular visits to a health professional.

Abraham and Sheeran [20] reported diverse applicability of the HBM to diseases, including hypertension control, alcohol, smoking, vaccination, diabetes, and psychiatry. Taking a cue from this diverse applicability of the HBM and previous studies such as Belay et al. [21], Nyaaba and Akurugu [22], the current study used age, gender, and awareness to predict cancer screening.

## 2 Materials and Methods

### 2.1 Study Design

This study employed an online cross‐sectional survey. The questionnaire presented items to measure the cancer awareness, gender, age, and screening behavior of participants.

### 2.2 Study Setting

The online survey was conducted in several Ghanaian cities namely, Accra, Tema, Kumasi, Cape Coast, Ho, Tamale, Takoradi, and Sunyani. These are regional capitals and are the most affluent cities in Ghana with a high rate of internet penetration and active cancer outreach campaigns. Based on projections by the Ghana Statistical Service, the estimated adult literate population of these cities is 6000 000 [23]. The regional capital status of these cities also provides them with Regional Hospitals capable of screening for most of the common cancers identified in Ghana. Additionally, there are numerous private health facilities with cancer screening services in these cities. Cancer awareness is higher in these “metropolitan” areas because of active NGOs and government campaigns aiming to increase awareness and screening. Survey links were sent by the researchers to the participants between June and August 2021. To prevent individuals from answering twice, they received the links once and after completion they informed the individual who recruited them. These recruiters did not reveal the identities of the respondents to the researchers.

### 2.3 Participants

To be included in this study, participants were required to be adult, have internet access and be resident in Accra, Tema, Kumasi, Cape Coast, Ho, Tamale, Takoradi or Sunyani, all in Ghana. After meeting these criteria, the online questionnaire presented a question asking the individuals whether they were interested in completing the survey online before links were sent to them. The first batch of 100 participants were randomly recruited from the contacts of the researchers and universities in the above‐named cities. Subsequently the snowball technique was used to recruit 407 more participants.

### 2.4 Variables

The outcome variable in this study was cancer‐screening behavior. Cancer awareness, gender, and age were predictor variables. Cancer screening behavior was measured as a dichotomous variable with participants indicating whether they had a history of cancer screening. To measure cancer awareness, participants were asked whether they knew about breast cervical, colon, stomach, liver, prostate and ear, nose, and throat or ENT cancers. Gender was a dichotomous variable, and participants could only select either female or male. Age was measured on a continuous scale with participants stating how old they were at the time of the study. These variables were used to predict cancer‐screening habits because institutions such as the Ghana Health Service, Ghana's Ministry of Health, and NGOs organize campaigns to create awareness of these cancers. These campaigns target specific genders for some cancers and encourage older people to be screened. This observation and support from research make these three variables important in the prediction of screening habits.

### 2.5 Data Sources/Measurements

Data collection: An online survey was deployed on google forms. All data were downloaded and analyzed with SPSS.

Data collection tool: The questionnaire was in three sections. These were cancer awareness, screening history and demographics. The demographics sections collected information about age and gender while the screening history section presented two items which asked participants “Have you ever screened for any cancer?” and “If you have screened for cancer, which type?”. To measure awareness, seven cancers, namely, breast, cervical, colon, stomach, liver, prostate and ear, nose, and throat or ENT were listed. Participants were asked “Have you ever heard of the following?”. The use of single item measures for some key variables such as awareness and screening habit is supported by previous studies such as Williams [24] and Bowling [25].

### 2.6 Bias

To avoid the confounding effects of professional cancer knowledge from medical professionals, the research widely distributed the questionnaire to non‐medical professionals.

### 2.7 Sample Size

The study size was derived from a random sampling of adults who were on various WhatsApp groups of the researchers and their contacts. The sample size was computed based on the estimated population of adults who were literate and had access to the internet in the cities covered by the study. Using the estimated population of 6000 000, a 95% confidence interval and a 5% margin of error, the required sample size was 385. However, due to the researcher's data collection efforts and the resounding interest of the target population in the study, 507 responses were recorded. The target population of literate adult internet users was chosen because of the nature of current cancer awareness campaigns and observations. Current campaigns mostly use English and deploy campaigns through multiple channels including the internet and offices. The researchers have additionally observed from clinical practice that, most patients who request screening services are literate and exposed to cancer awareness messages.

### 2.8 Quantitative Variables and Statistical Methods

This statistical approach for this study was quantitative. Both univariate and multivariate analysis were conducted. To ensure consistency and data quality only complete responses were included in the final dataset. This data set was analyzed with version 26 of IBMs SPSS. Univariate analysis (see Table 1) presented the frequencies and percentages of variables. To ensure that all variables to be included in the data were not perfectly correlated and to reveal the strength of relationships, a spearman rho correlation analyses (see Table 2) was conducted. This computation included age, gender, cancer awareness, and screening habit. A model which tested the hypothesis that cancer awareness, age, and gender predict cancer‐screening habits was tested with a logistic regression (see Table 3).

**TABLE 1:** Descriptive table of responses and demographics.

Variable	Frequency	Percentage
Previous screening (*N* = 503)		
No	314	62.4
Yes	189	37.6
Awareness		
Breast cancer (*N* = 503)		
No	2	0.4
Yes	501	99.6
Cervical cancer (*N* = 503)		
No	19	3.8
Yes	484	96.2
Colon cancer (*N* = 500)		
No	64	12.8
Yes	436	87.2
Ear, nose and throat cancers (*N* = 499)		
No	157	31.5
Yes	342	68.5
Gastric cancer (*N* = 499)		
No	114	22.8
Yes	385	77.2
Liver cancer (HCC*) (*N* = 500)		
No	67	13.4
Yes	433	86.6
Prostate cancer (*N* = 503)		
No	17	3.4
Yes	486	96.6

Abbreviation: HCC* = hepatocellular carcinoma.

**TABLE 2:** Table of correlations for age, cancer awareness, gender, and cancer screening.

Variable	1	2	3	4
1. Age	—			
2. Awareness	0.09*	—	‐.	
3. Gender	0.06	−0.09	—	
4. Cancer screening	0.28*	0.07	−0.43*	—

*Note:* *Correlation significant (*p* < 0.05).

**TABLE 3:** Model statistics of logistic regression with cancer screening as the outcome variable.

Variable	*B*	SE	Wald	Exp (*B*)	Sig	95% CI for Exp (*B*)	
						Lower	Upper
Age	0.10	0.01	52.65	1.11	0.00	1.08	1.14
Awareness	0.00	0.09	0.00	1.00	0.99	0.85	1.18
Gender	−2.71	0.30	80.36	0.07	0.00	8.32	27.22
Constant	−0.35	0.69	0.26	0.71	61		

*Note:* Sig = *p* value.

Abbreviation: SE = standard error.

### 2.9 Ethical Considerations

The study adhered to the ethical principles of anonymity, confidentiality, and informed consent. To maintain anonymity, personal identifying information such as names, email, phone numbers, and signatures were not collected. Researchers assured participants that all responses were confidential and there was no way to trace their responses to them. Additionally, data from the study was kept on secure cloud servers accessible only to the researchers. Participants were required to agree to the informed consent statement before they could proceed with participation. To maintain anonymity, participants were not required to sign or type their names on the statement. Any participant who disagreed with the informed consent had the option to refuse participation in the study. Participation therefore meant the participant has agreed to the informed consent statement. Ethical approval for this project was granted by the CCTH IRB with a Federal wide Assurance Number (FWA) of IRB00014450 and a reference number of CCTHERC/EC/2024/189.

## 3 Results

### 3.1 Participants

A total of 507 responses were received but only 503 were included in the final statistical analysis. Four responses were excluded because they were incomplete. The participants were aged between 18 and 74 with a mean age of 32.74 and a standard deviation of 9.10. The responses were received from 309 females, which accounted for 61.4% of responses and 194 males, accounting for 38.6%.

### 3.2 Descriptive Data

A breakdown of the data on age showed that 48.6% (244) of respondents were aged 19–30, 31.5% (158) were 31–40, and 19.9% (100) were 41–74 years old. Responses indicated that most of the participants were aware of the cancers listed in our research but had never been screened for any of them (see Table 1).

### 3.3 Outcome Data

In the two‐stage multivariate analysis, a correlation treated all variables the same and a logistic regression used cancer‐screening habits as the outcome. The first stage tested for perfect correlations and the relationship between age, cancer awareness, cancer screening habits, and gender. In the second stage, cancer‐screening habits was used as an outcome, in line with the objectives of this study.

### 3.4 Main Results

#### 3.4.1 Relationship Between Age, Cancer Awareness, Gender, and Cancer Screening

Spearman rank correlation coefficients were computed to test the relationship between age, cancer awareness, gender, and cancer screening habit in paired comparisons (see Table 2).

#### 3.4.2 Cancer Screening Habit Is Predicted by Age, Cancer Awareness, and Gender

The prediction was supported for age and gender with cancer screening habit as the outcome. In the logistic regression model, age and gender predicted cancer‐screening habits. Cancer awareness did not predict cancer‐screening habit (see Table 3). The predictive strength and directions of age and gender in the model were signaled by the spearman rho correlation computed before the logistic regression.

### 3.5 Summary of Results

In summary, the descriptive results point to a high level of awareness and low screening uptake. Respondents were aged between 19 and 74 (mean = 32.74, standard deviation = 9.10). The age range was appropriate because of the list of cancers considered. Gender and age predicted screening habits. Results indicated that awareness did not predict screening habits. These results are discussed in the next section.

## 4 Discussion

### 4.1 Key Results

Discussing the hypotheses, the first notable finding is the support for the role of gender in predicting screening habits. In this study, females reported more active screening habits than males. This may be because of the relatively consistent campaign to convince females to screen for breast and cervical cancers [4]. Entire months of the year are dedicated to breast and cervical cancer awareness programs. Again, there are many advocacy groups run by female survivors of these cancers that create awareness and encourage screening. With the possibility of self‐screening for human papilloma virus, screening for cervical cancer has invariably become patient centered and thus improved compliance and uptake of cervical cancer screening. These in addition to the WHO's call for elimination of cervical cancer may have contributed to the increased awareness and higher screening practices among women. Comparatively, campaigns for men have focused on Prostate cancer screening from the age of 40 and this makes age an influential factor in the habits of men. Unfortunately, though liver cancer is a leading cause of cancer death and is associated with known causes of chronic liver disease such as hepatitis B and C virus infection, chronic alcohol abuse and aflatoxin infection, only few campaigns are ongoing about the prevention and early detection of liver cancer. The same scenario plays out for gastrointestinal cancers (stomach and colon).

In the second finding, age predicted screening habits. Campaigns for cancer screening have signaled an increased risk with age especially for prostate cancer. This may have led to increased likelihood of screening as one gets older, especially in males. It is thus not a surprising finding. The results suggest that the likelihood of screening increases with age and older people are more likely to screen for multiple cancers. As mentioned by some studies such as Shirazu et al. [7], most cancer screening campaigns in Ghana advocate screening for older people. For instance, for prostate cancer screening, men are advised to screen after the age of 50 while women are advised to start screening after age 25 for cervical cancer. Similarly, for breast cancer, women are mostly advised to screen later in their 40s.

The third finding, which suggested that cancer awareness, did not predict the likelihood of screening, was surprising. The finding may however be explained by several barriers which can impede screening as identified by Ampofo et al. [9] and Ebu et al. [6]. While individuals may be aware of the need to screen many face challenges accessing screening centers and funding the procedure. This maybe a hurdle which many have not been able to surmount. Again, many countries where screening has been successful run a central call and reminder system that ensures that patients are reminded of screening schedules and get scheduled for screening at a health facility near them. Furthermore, in those countries, the cost of screening and treatment for detected lesions is borne by the state, ensuring that beneficiaries do not have to worry about cost. The absence of these factors may negatively impact the screening habits of our respondents.

In summary, findings suggest that age and gender predict screening behavior better than awareness. The researchers can speculate that the reason for the lack of a relationship between awareness and screening could be barriers such as unavailability of affordable screening centers and the absence of an organized screening reminder system.

### 4.2 Limitations

Despite the predictions by age and gender, the level of screening was still lower than 40% for the respondents. This could be attributed to the fact that a majority of our respondents are younger than 40 years although it was anticipated that this trend would be balanced by a higher representation of women given that cervical cancer and breast screening typically start almost a decade earlier than age 40. Therefore, the findings can only be generalized to adults around the mean age of 32. Additionally, the recommendations about increasing access to screening may be too general because the study focused on screening behavior and not barriers faced by respondents.

Further studies with larger and less skewed population may reveal the peculiarities of screening habits in different age groups.

### 4.3 Interpretation

Despite several publicity campaigns, cancer screening has not reached the numbers desired by healthcare stakeholders [9]. In the current paper, screening habits were low, though cancer awareness was relatively high. The increasing risk and disease burden posed by other cancers like colon, ENT, gastric, and liver cancer needs to be dealt with by the healthcare system. It was thus positive to note that our respondents were becoming more aware of the diverse nature of cancer and its ability to occur in other parts of the human body. Studies, such as that by O'Mahony et al. [15], have supported the important role of age and gender in predicting screening habits, a suggestion that is also upheld by findings in this study. These also conform to suggestions by the HBM [17].

Findings from this study have two clinical implications. First, for public health practice, findings imply that cancer awareness messages must be improved to include messages about screening based on age and gender. This is with regards to its importance and where screening centers can be found. Second, for clinicians offering screening services, findings imply that these services should be publicized, and practitioners should collaborate with public health officials to improve the visibility.

### 4.4 Generalizability

This study included most of the commonly diagnosed cancers in Ghana. These cancers exert a significant toll on both personal and public health budgets in Ghana and other African countries. Participants were selected from the biggest cities in Ghana where prevention and early detection campaigns are more frequent, but morbidity and mortality continue to rise. The scope of this study permits an extension of findings to cancer screening campaigns for both genders in cities where screening facilities are available. These include both private and public screening centers as well as periodic screening events, which run for months and are dedicated to specific cancers such as the breast cancer. Findings can help practitioners understand the scope of awareness, its impact on screening and the roles played by both gender and age. Additionally, the findings can help practitioners and researchers understand the current gap between cancer awareness campaigns and screening habits.

### 4.5 Conclusion

Age and gender play important roles in predicting screening habits in relation to the broader scope of cancers sampled. Older individuals and women are more likely to screen for cancer.

Awareness did not predict screening habits.

The study extends suggestions that screening needs to be a core part of campaigns by healthcare interest groups. We recommend that efforts that improve the ease and likelihood of screening should be intensified.

## Author Contributions

**Patrick Kafui Akakpo:** conceptualization (equal), data curation (supporting), investigation (lead), methodology (supporting), project administration (equal), supervision (lead), writing – original draft (supporting), writing – review and editing (lead). **Martin Gameli Akakpo:** conceptualization (equal), data curation (lead), formal analysis (lead), investigation (supporting), methodology (supporting), project administration (equal), writing – original draft (lead), writing – review and editing (supporting).

## Conflicts of Interest

The authors declare no conflicts of interest.

## Data Availability

The data file used for this paper is available upon request.
